# Organoarsenic Compounds with In Vitro Activity against the Malaria Parasite *Plasmodium falciparum*

**DOI:** 10.3390/biomedicines8080260

**Published:** 2020-08-02

**Authors:** Sofia Basova, Nathalie Wilke, Jan Christoph Koch, Aram Prokop, Albrecht Berkessel, Gabriele Pradel, Che Julius Ngwa

**Affiliations:** 1 Division of Cellular and Applied Infection Biology, Institute of Zoology, RWTH Aachen University, Worringerweg 1, 52074 Aachen, Germany; sofia.basova@rwth-aachen.de (S.B.); pradel@bio2.rwth-aachen.de (G.P.); 2 Department of Paediatric Oncology, Children’s Hospital Cologne, Amsterdamer Straße 59, 50735 Cologne, Germany; nathalea2@web.de (N.W.); aram.prokop@helios-gesundheit.de (A.P.); 3 Department of Chemistry, Organic Chemistry, University of Cologne, Greinstraße 4, 50939 Cologne, Germany; koch@jc-koch.de (J.C.K.); berkessel@uni-koeln.de (A.B.); 4 Department of Paediatric Oncology, Helios Hospital Schwerin, Wismarsche Strasse 393-397, 19049 Schwerin, Germany

**Keywords:** organoarsenic compound, antimalarial, gametocytocidal, malaria, transmission, *Plasmodium falciparum*

## Abstract

The rapid development of parasite drug resistance as well as the lack of medications targeting both the asexual and the sexual blood stages of the malaria parasite necessitate the search for novel antimalarial compounds. Eleven organoarsenic compounds were synthesized and tested for their effect on the asexual blood stages and sexual transmission stages of the malaria parasite *Plasmodium falciparum* using in vitro assays. The inhibitory potential of the compounds on blood stage viability was tested on the chloroquine (CQ)-sensitive 3D7 and the CQ-resistant Dd2 strain using the Malstat assay. The most effective compounds were subsequently investigated for their effect on impairing gametocyte development and gametogenesis, using the gametocyte-producing NF54 strain in respective cell-based assays. Their potential toxicity was investigated on leukemia cell line Nalm-6 and non-infected erythrocytes. Five out of the 11 compounds showed antiplasmodial activities against 3D7, with half-maximal inhibitory concentration (IC_50_) values ranging between 1.52 and 8.64 µM. Three of the compounds also acted against Dd2, with the most active compound As-8 exhibiting an IC_50_ of 0.35 µM. The five compounds also showed significant inhibitory effects on the parasite sexual stages at both IC_50_ and IC_90_ concentrations with As-8 displaying the best gametocytocidal activity. No hemolytic and cytotoxic effect was observed for any of the compounds. The organoarsenic compound As-8 may represent a good lead for the design of novel organoarsenic drugs with combined antimalarial and transmission blocking activities.

## 1. Introduction

The tropical disease malaria is a major health threat with an estimated 228 million cases and 405,000 deaths in 2018 [1]. Chemotherapeutic measures are increasingly encountering resistance of the *Plasmodium* parasites to current antimalarial regimes, including the artemisinin-based combination therapies, which serve as first line drugs for the treatment of malaria tropica. Most antimalarials are active mainly on the asexual blood stages, which are responsible for the clinical manifestation of the disease, but not on the gametocyte stages, which are important for disease transmission from the human to the mosquito. Primaquine is the only drug with significant gametocytocidal effect but it is not widely used because of its hemolytic toxicity in individuals with glucose-6-phosphate-dehydrogenase deficiency [2]. There is therefore the need to search for new drugs with combined activities against the blood and transmission stages.

Arsenic is a toxic metalloid commonly present in many minerals as well as in food, water, soil and the air [3,4]. Particularly inorganic arsenic compounds are highly toxic and comprise numerous valence states including arsenic trioxide (As_2_O_3_) and realgar [5]. In contrast, organoarsenic compounds are less toxic than their inorganic counterpart and a variety of them are found naturally in the environment because of bio-methylation and other biosynthetic pathways [6]. Arsenic compounds have been shown to display good therapeutic potentials and have been used in traditional medicine for the treatment of diseases such as skin cancer and fevers [5]. Studies have shown that compounds which inhibit cancer growth also often exhibit an inhibitory effect against the malaria parasite and vice versa [7,8]. In addition, synthetic organoarsenic drugs have been used for the treatment of protozoan infections caused by *Trypanosoma brucei*, *Trichomonas vaginalis* and *Entamoeba histolytica*, but most of the drugs were later abandoned due to their side effects. It should be noted that the malaria parasite is a member of the protozoans, indicating that it could be targeted by arsenic compounds.

In this study, we evaluated the antiplasmodial activity of newly designed organoarsenic compounds, together with several known organoarsenic compounds against the blood and sexual stages of *P. falciparum* by in vitro assays.

## 2. Materials and Methods 

### 2.1. Organoarsenic Compound As-1–As-11 Synthesis

**Figure 1:**
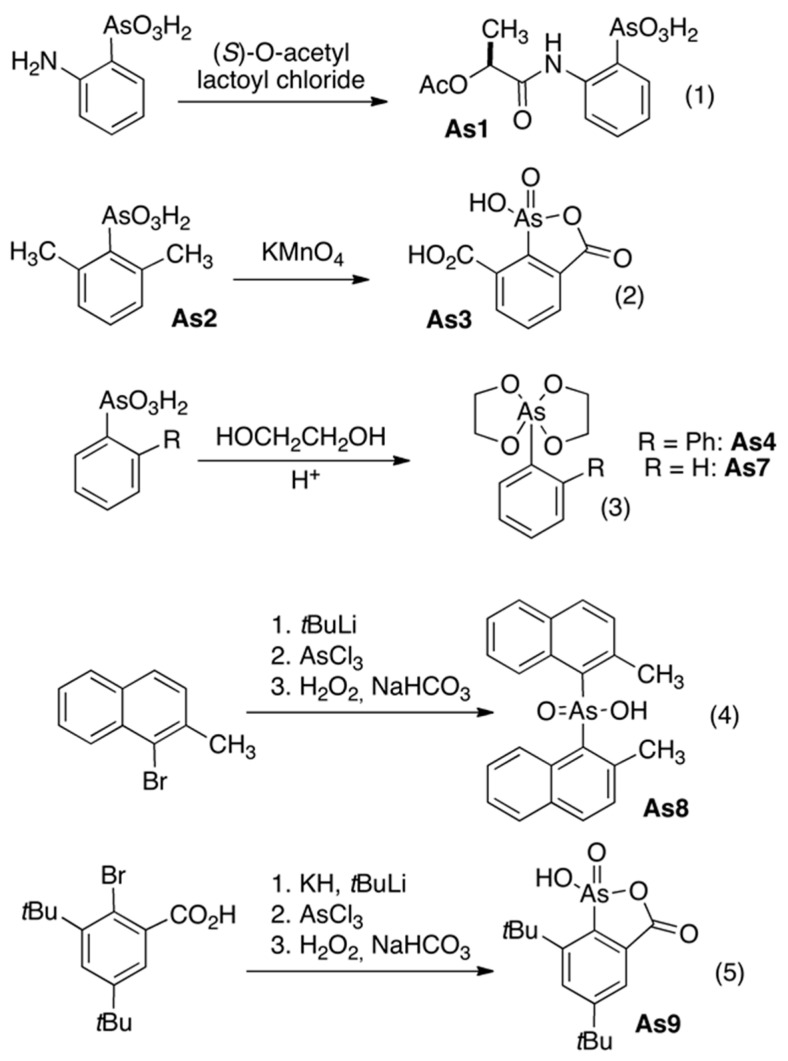
Figure showing the reactions used for the synthesis of organoarsenic compounds.

**Figure 2:**
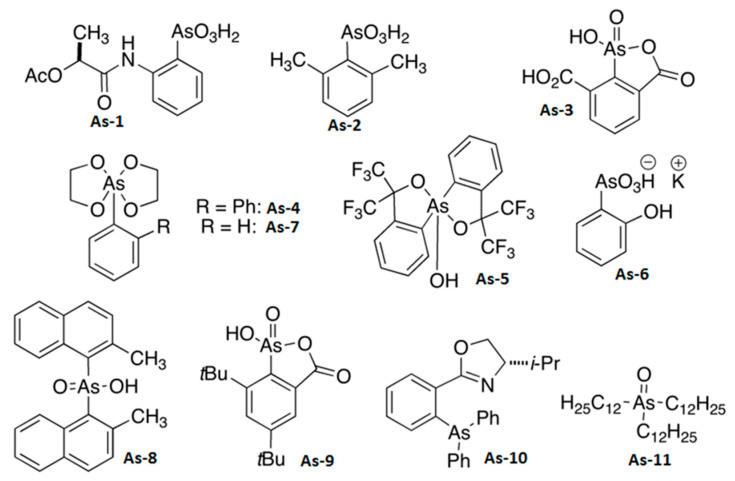
Chemical structure of synthesized organoarsenic compounds tested in the study.

**Figure 3:**
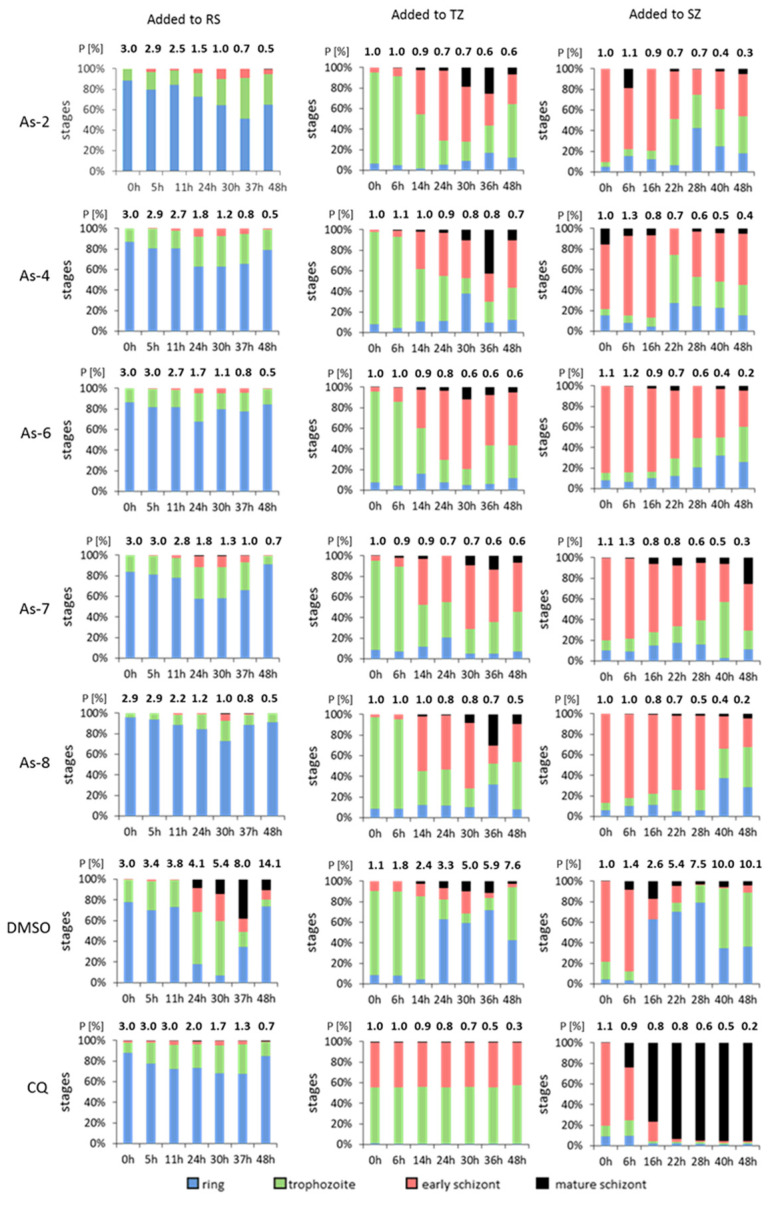
Stage-specific inhibition of intraerythrocytic development of *P. falciparum* by organoarsenic compounds. Compounds at IC_90_ concentrations were added to the ring stages (3% parasitemia), trophozoite and early schizont stages (1% parasitemia). Smears were prepared at seven time points between 0 and 48 h and the numbers of ring stages, trophozoites, early schizonts, and mature schizonts were counted via Giemsa smears. Then, 0.5% *v*/*v* DMSO was used as a negative control and CQ (0.25 µM) served as a positive control. Fifty parasites were counted on each slide. The experiment was performed four times in triplicate. RS, ring stage; P, parasitemia; TZ, trophozoite; SZ, schizont.

**Figure 4:**
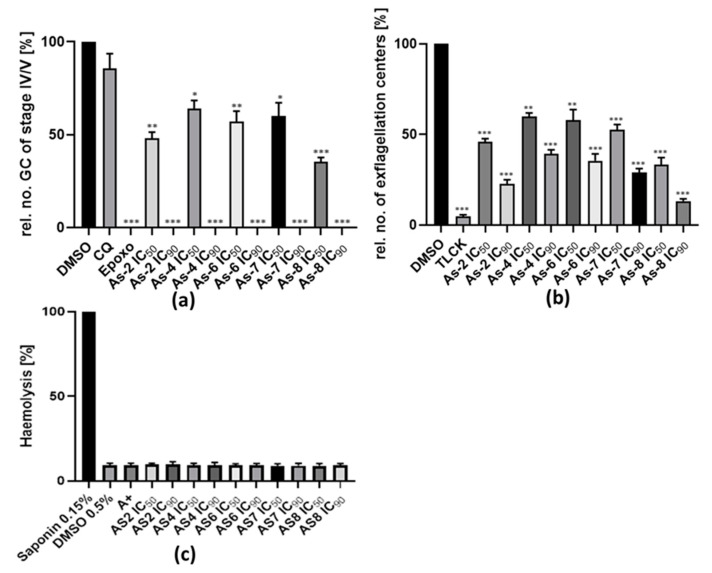
Effect of organoarsenic compounds on gametocyte development, male gametogenesis and erythrocyte viability. (**a**) Effect of organoarsenic compounds on gametocyte development. Stage II gametocyte-rich cultures were treated with IC_50_ and IC_90_ concentrations of compounds for 2 d and further cultivated for another 7 d. The numbers of stage IV and V gametocytes were counted and normalized to 0.5% *v*/*v* dimethyl sulfoxide (DMSO) treatment (set to 100%). CQ (0.25 µM) was used as a negative control and epoxomicin (50 nM) served as a positive control. (**b**) Inhibition of exflagellation by organoarsenic compounds. Compounds at IC_50_ and IC_90_ concentrations were added to mature gametocyte cultures for 15 min prior to their activation with xanthurenic acid. The numbers of exflagellation centers were counted at 15 min post-activation and normalized to 0.5% *v*/*v* DMSO (set to 100%). Tosyl-l-lysyl-chloromethane hydrochloride (TLCK) (30 µM) was used as positive control. (**c**) Hemolytic effect of organoarsenic compounds. Compounds at IC_50_ and IC_90_ concentrations were added to fresh erythrocytes (5% hematocrit) and incubated at 37 °C for 48 h. The absorbance of free hemoglobin in the supernatant was measured at OD_550_ nm and normalized to 0.15% *w*/*v* saponin (set to 100%), which was used as positive control in the experiments. The 0.5% DMSO and A+ medium served as a negative control. The graphs represent the results of three to four independent experiments performed in triplicate (mean ± SEM; standard error of mean). Asterisks represent a significant difference between the tested compounds and DMSO control, *** *p* < 0.001; ** *p* < 0.01; * *p* < 0.05 (student’s t-test).

The synthesis of the organoarsenic compounds As-1–4 and 7–9 is summarized in Figure 1. Compound As-1 was prepared by acylation of *ortho*-arsanilic acid with the acid chloride of (*S*)-*O*-acetyl lactic acid (Figure 1, (1); see Supplementary Information 1, Figure S1 for experimental details, compound characterization, and X-ray crystal structure). The 2,6-Dimethylphenyl arsenic acid (As-2) was prepared by *Bart*-reaction from 2,6-dimethylaniline, as described in the literature [9] (see Supplementary Information 2, Figure S2 for experimental details, compound characterization, and X-ray crystal structure). The mixed anhydride As-3 was prepared from the arsenic acid As-2 by oxidation with potassium permanganate (Figure 1, (2); see Supplementary Information 3, for experimental details and compound characterization). The ester As-4 was obtained by reaction of biphenyl 2-arsenic acid with ethylene glycol (Figure 1, (3)); analogous to the method used for the preparation of compound As-7 (Figure 1, (3)). The arsenic acid derivative As-5 was prepared as described in [10]. The potassium salt of *ortho*-hydroxyphenyl arsenic acid (As-6) was prepared as described in [11]. The arsenic acid ethylene glycol diester As-7 (Figure 1, (3)) was prepared according to [12]. The arsenic acid As-8 was prepared from 1-bromo-2-methylnaphthalene by lithiation and reaction with AsCl_3_/H_2_O_2_ (Figure 1, (4); see Supplementary Information 4, Figure S3 for experimental details, compound characterization, and X-ray crystal structure). The mixed anhydride As-9 was prepared from 2-bromo-3,5-di-tert.-butylbenzoic acid by the method described for As-8 (Figure 1, (5); see Supplementary Information 5, Figure S4 for experimental details, compound characterization, and X-ray crystal structure). The arsane As-10 was prepared according to [13]. The arsane oxide As-11 was prepared according to [14].

### 2.2. Parasite Culture

The *P. falciparum* CQ-sensitive strain 3D7 and CQ-resistant strain Dd2 were cultured with an RPMI 1640/HEPES cell culture medium (Gibco, Thermo Scientific, Waltham, USA) supplemented with 10 µg/mL gentamicin (Gibco Thermo Scientific, Waltham, USA), 50 µg/mL hypoxanthine (Sigma-Aldrich, St. Louis, Missouri, USA) and 0.5% *v*/*v* Albumax II (Gibco, Thermo Scientific, Waltham, USA) at 5% hematocrit. To generate gametocytes, the *P. falciparum* NF54 strain was cultivated in the RPMI medium in the presence of 10% inactivated human serum [15]. Cultures were maintained at 37 °C in an atmosphere of 5% CO_2_, 5% O_2_ and 90% N_2_. To synchronize the culture, parasites with 5% ring stages were centrifuged, the pellet was resuspended in five times pellet volume of 5% *w*/*v* sorbitol/dd H_2_O and incubated for 10 min at room temperature (RT) [16]. Cells were washed once with RPMI to remove sorbitol and further cultivated as described above. Human serum and erythrocyte concentrate were obtained from the Department of Transfusion Medicine, University Hospital Aachen, Germany. Donor sera and blood samples were pooled and kept anonymous. The work with human blood was approved by the Ethics commission of the Rheinisch-Westfälische Technische Hochschule (RWTH) University Hospital (EK 007/13).

### 2.3. Malstat Assay

To determine the inhibitory effect of the organoarsenic compounds on the *P. falciparum* erythrocytic replication, the Malstat assay was used as described [17,18,19]. Synchronized ring stage cultures of *P. falciparum* strains 3D7 and Dd2 were plated in triplicate in 96-well plates (200 µL/well) at a parasitemia of 1% in the presence of the compounds dissolved in 0.5% *v*/*v* dimethyl sulfoxide (DMSO; Sigma Aldrich, Taufkirchen, Germany) at concentrations ranging from 200 µM to 2.6 nM. Chloroquine (CQ; Sigma Aldrich, Taufkirchen, Germany) was used as an internal control in the experiments, while parasites incubated with 0.5% *v*/*v* DMSO served as a negative control. The parasites were incubated with the compounds for 72 h at 37 °C in the presence of 5% O_2,_ 5% CO_2,_ and 90% N_2_. Afterwards, 20 µL was removed and added to 100 µL of the Malstat reagent (0.1 % Triton X-100 (Carl Roth, Karlsruhe, Germany), 10 mg of L-lactate (Sigma Aldrich, Taufkirchen, Germany, 3.3 mg Tris (Carl Roth, Karlsruhe, Germany) and 0.33 mg of APAD (3-Acetylpyridine adenine dinucleotide; Sigma Aldrich, Taufkirchen, Germany) dissolved in 1 mL of distilled water, pH 9.0) in a new 96-well microtiter plate (Hartenstein, Würzburg, Germany). The plasmodial lactate dehydrogenase (LDH) activity was then assessed by adding a 20 μL mixture of nitro blue tetrazolium (NBT (F); Roche, Basel, Switzerland)/Diaphorase;(Sigma Aldrich, Taufkirchen, Germany) 1:1; 1mg/mL stock each to the Malstat reaction. The optical densities were measured at 630 nM and the IC_50_ values were calculated from variable-slope sigmoidal dose–response curves using the GraphPad Prism program version 5 (GraphPad Software Inc., La Jolla, CA, USA).

### 2.4. Stage-Specific Inhibition Assay

To determine the stage at which the compounds are active during erythrocytic replication, stage-specific inhibition assays were carried out. Compounds at IC_90_ concentrations were added to highly synchronized ring stages (3% parasitemia), trophozoites (1.5% parasitemia) and schizonts (1.5% parasitemia) and incubated at 37 °C. Giemsa-stained smears were prepared at seven time points between 0 and 48 h of incubation. The numbers of ring, trophozoite, and early and mature schizont stages were counted and the parasitemia determined at each time point. Fifty parasites were counted each in triplicate. CQ at 0.25 µM was used as a positive control and 0.5% *v*/*v* DMSO as a negative control.

### 2.5. Gametocyte Toxicity Assay

To investigate the gametocytocidal activities of the compounds, the *P. falciparum* gametocyte-producing strain NF54 was grown at high parasitemia to induce gametocytogenesis. Upon the appearance of stage II gametocytes in the culture, 1 mL of the culture was aliquoted in triplicate in a 24-well plate (Hartenstein, Würzburg, Germany) in the presence of compounds at IC_50_ and IC_90_ concentrations. The gametocytes were treated with the compound (As-2, As-4, As-6, As-7, and As-8) for 2 d and subsequently cultured in regulator medium, which was replaced daily. On day 10, samples were taken, Giemsa-stained smears were prepared, and the gametocytemia was evaluated by counting the numbers of gametocyte stages IV and V in a total number of 1000 erythrocytes in triplicate.

### 2.6. Exflagellation Inhibition Assay

To determine the effect of the compounds on male gametogenesis, 100 µL of mature gametocyte cultures were pre-incubated with the compounds (As-2, As-4, As-6, As-7, and As-8) at IC_50_ and IC_90_ concentrations for 15 min at 37 °C. Gametogenesis was induced in vitro by incubating mature gametocyte cultures in 100 µM xanthurenic acid (Sigma Aldrich, Taufkirchen, Germany) dissolved in 1% *v*/*v* 0.5 M NH_4_OH/dd H_2_0 for 15 min at RT [20,21]. The mixture was briefly centrifuged, about 85 µL of medium was carefully removed and the pellet was resuspended in an equal amount of the remaining medium. Approximately 10 µL was then placed on a glass slide (Hartenstein, Würzburg, Germany) and the numbers of exflagellation centers were counted in 30 optical fields in triplicate using a Leica DMLS microscope at 400-fold magnification. The inhibition of exflagellation was calculated as a percentage of the number of exflagellation centers in treated cultures in relation to the number of exflagellation centers in 0.5% *v*/*v* DMSO, which was set to 100%. TLCK (Tosyl-l-lysyl-chloromethane hydrochloride; Carl Roth, Karlsruhe, Germany) at 30 µM was used as a positive control in the experiments.

### 2.7. Hemolysis Assay

To assess if the compounds lyse non-infected erythrocytes, a hemolysis assay was performed. Non-infected red blood cells were resuspended in the cell culture medium at a 5% hematocrit, plated in triplicate in a 96-well microtiter plate and incubated with the compounds (As-2, As-4, As-6, As-7, and As-8) at IC_50_ and IC_90_ concentrations. Erythrocytes incubated in the cell culture medium supplemented with 0.15% *w*/*v* saponin were used as a positive control, while 0.5% *v*/*v* DMSO served as a negative control. The erythrocyte cultures were incubated at 37 °C for 48 h. After incubation, the plates were centrifuged at 800 g for 2 min and 100 μL of the supernatant was transferred from each well to a new 96-well microtiter plate. The optical densities were measured at 550 nM.

### 2.8. Cytotoxicity Assay

The cytotoxicity of the compounds was evaluated by measuring the LDH release in the leukemia cell line Nalm-6 after 1 h of incubation (37 °C, 5% CO_2_) with the compounds (As-2, As-4, As-6, As-7, and As-8) at concentrations of 1–100 µM. LDH activity in the supernatant was determined using the Cytotoxicity Detection Kit (Roche, Mannheim, Germany). After centrifugation at 350 g for 5 min, 20 µL of the cell-free supernatant were diluted with 80 µL phosphate-buffered saline (PBS). Subsequently, 100 µL of the reaction solution containing 2-[4-idophenyl]-3-[4-nitrophenyl]-5-phenyltetrazolium chloride (INT), sodium lactate, NAD+ and diaphorase were added for the coupled enzymatic test. The time-dependent formation of formazan salt was photometrically quantified at 492 nm. As a positive control, 0.1% Triton X-100 (Sigma Aldrich, Taufkirchen, Germany) in culture medium was used for cell lysis and measured values represented 100% cytotoxicity. Untreated cells and DMSO control served as negative controls for background extinction as well as for the determination of 100% cell viability.

## 3. Results

### 3.1. Organoarsenic Compounds

Figure 2 summarizes the chemical structures of the organoarsenic compounds As-1 to As-11 successfully synthesized and used in this study. Compounds As-1,2,6 are arsenic acids, As-4,5,7 are esters of arsenic acids, while As-3,9 are mixed anhydrides of arsenic and carboxylic acids. Compound As-8 is an arsenic acid, while compounds As-10 and As-11 represent examples of an arsane and an arsane oxide, respectively. The details of the chemical synthesis of compounds As-1 to As-11 can be found in Section 2.1 and supplementary File 1.

### 3.2. The Organosarsenic Compounds Exhibit Antimalarial Activity

The 11 organoarsenic compounds (As-1 to As-11) were first tested for their antiplasmodial activities against the CQ-sensitive 3D7 strain of *P. falciparum*. After incubating the parasites for 72 h with a serial dilution of the compounds ranging from 200 µM to 2.6 nM, the viability of the parasites was assessed using the Malstat assay, which measures *P. falciparum*-specific lactate dehydrogenase activity. The half-maximal inhibitory concentration (IC_50_) and the 90% inhibitory concentration (IC_90_) were calculated for each compound. CQ was used as positive control in the assays.

We observed that five out of the 11 compounds, As-2, As-4, As-6, As-7 and As-8 displayed antiplasmodial activities with IC_50_ values at lower micromolar ranges (Table 1; Figure S5). Four other compounds, As-5, As-9, As-10 and As-11, showed antiplasmodial activity against 3D7 at higher micromolar ranges, while As-1 and As-3 were inactive.

The five most active compounds were then tested against the CQ-resistant Dd2 strain. As-2 and As-6 maintained their inhibitory activities against Dd2; As-8 displayed increased activities with an IC_50_ = 0.35 ± 0.099 µM. As-4 and As-7 showed reduced antiplasmodial activities (Table 1; Figure S6). As expected, CQ had a 100-fold decrease in activity against Dd2 compared to 3D7.

### 3.3. Organoarsenic Compounds Inhibit Different Asexual Blood Stages of the Malaria Parasite

To obtain more information on the specific intraerythrocytic stages that are impaired in their development by the organoarsenic compounds, we investigated the inhibitory effect via Giemsa-stained blood-stage quantification. The five most-active compounds As-2, As-4, As-6, As-7 and As-8 were added to synchronized ring stages, trophozoites, or schizonts at their respective IC_90_ concentrations. CQ (0.25 µM) and DMSO (0.5% *v*/*v*) were used as positive and negative controls, respectively. Giemsa smears were prepared at seven time points between 0 and 48 h following treatment. The numbers of rings, trophozoites, early schizonts, and mature schizonts were microscopically counted and the parasitemia at each time point was determined.

Parasites treated with DMSO underwent the 48-h intraerythrocytic replication cycle and the parasitemia increased five to ten-fold, depending on the intraerythrocytic stage at time zero (Figure 3). As expected, CQ treatment killed the parasites rapidly. No progression in intraerythrocytic stage development could be observed when CQ was added to the ring or trophozoite stage, while the parasitemia decreased by approximately two-thirds. When CQ was added to early schizonts, these were able to mature, but no further progression to the next generation cycle was observed.

When the five compounds were added to the ring stages, these did not develop any further and the parasitemia decreased by more than 80% (Figure 3; Figure S7). A similar decrease in parasitemia was also observed when the compounds were added to the trophozoite and schizont stages, but the surviving parasites could further develop to the early schizont stages before progression stopped. These data indicate that the organoarsenic compounds are most active during early intraerythrocytic development, when the parasites grow in size before initiation of nuclear division.

### 3.4. Treatment with Organoarsenic Compounds Affects Gametocyte Development

To eradicate malaria, it is important for a drug to be effective against both the asexual blood stages and the sexual stages, which are responsible for the transmission of the disease from human to human by the mosquito. We therefore investigated if the organoarsenic compounds affect the development of gametocytes from stage II to V. In this regard a gametocyte stage II-rich culture was treated with As-2, As-4, As-6, As-7 and As-8 at IC_50_ and IC_90_ concentrations for two days and the gametocytes were allowed to develop further for another seven days, before Giemsa smears were prepared. The proteasome inhibitor epoxomicin (60 nM) was used as a positive control, while CQ (0.25 µM) and DMSO (0.5% *v*/*v*) were used as negative controls in the experiments.

After the addition of the compounds at their IC_50_ concentrations, the numbers of stage IV and V gametocyte stages were reduced by approximately half for all compounds indicating that gametocyte maturation was affected (Figure 4a). As-8 had the highest gametocytocidal effect and reduced gametocyte development by about 60% at IC_50_ concentrations. The surviving gametocytes exhibited healthy morphologies (Figure S8). As reported before [17,22], treatment with epoxomicin killed all of the parasites. Treatment with DMSO, used for negative control, did not affect the gametocytes, while CQ only had a minor effect on gametocyte development, as described previously [2,17]. At IC_90_ concentrations, all gametocytes had disappeared, and only cell fragments were visible (Figure S8). Epoxomicin, which served as a positive control, resulted also in complete elimination of gametocytes while CQ at 0.25 µM reduced the number of gametocytes only by about 20%.

### 3.5. The Organoarsenic Compounds Inhibit Exflagellation

Another approach to test if a compound is able to block malaria transmission to the mosquito is to determine if male gametocyte exflagellation is inhibited. To determine if the compounds exhibit exflagellation inhibition activity in vitro, a mature gametocyte stage V culture was incubated with the five active compounds at IC_50_ and IC_90_ concentrations for 15 min at 37 °C. Subsequently, exflagellation was artificially induced by the addition of 100 µM xanthurenic acid at RT for 15 min. Afterwards, the number of exflagellation centers was microscopically counted and the numbers were compared to the ones in cultures incubated with 0.5 v/v DMSO. The cysteine/serine protease inhibitor TLCK, which is known to efficiently inhibit exflagellation [18,19,23,24], was used as a positive control in the assays. The results show a significant reduction in the numbers of exflagellation centers by approximately 50 and 80%, when the gametocytes were treated with the compounds at IC_50_ and IC_90_ concentrations, respectively (Figure 4b). The highest effect was obtained with As-8, which resulted in a 67% and 87% reduction in exflagellation at IC_50_ and IC_90_ concentrations, respectively. For comparison, treatment with TLCK reduced the numbers of exflagellation centers by 96%.

### 3.6. The Organoarsenic Compounds Exhibit No Hemolytic or Cytotoxic Effect on Human cells

To determine if the compounds would lyse human erythrocytes, a hemolysis assay was performed. Fresh erythrocytes were incubated with As-2, As-4, As-6, As-7, and As-8 at IC_50_ and IC_90_ concentrations for 48 h at 37 °C and hemolysis was determined by the amount of hemoglobin released in the cultures, as measured spectrometrically. Erythrocytes lysed with 0.15% saponin were used as positive control, while incubation of erythrocytes with 0.5% *v*/*v* DMSO or cell culture medium served as negative control. The assay demonstrated that the organoarsenic compounds had no hemolytic effect on the erythrocytes at IC_50_ and IC_90_ concentrations with no differences in extracellular hemoglobin content measurable between compound-treated and DMSO-treated cells (Figure 4c).

The compounds were further tested for their cytotoxic effect in the course of cell necrosis, using human Nalm-6 cells. The potential release of LDH from these cells after a 1-h incubation with the compounds at concentrations ranging from 1 to 100 µM. No noticeable cytotoxic effect could be detected for any of the compounds, including As-8 (Figure S9; Table 1).

## 4. Discussion

The discovery of new lead compounds targeting both the disease-causing asexual blood stages and sexual transmission stages represents an important step in the generation of effective anti-malarial drugs that are able to overcome the spread of parasite resistance and control spread of the disease. Despite large-scale screening programs, only few of such compounds have hitherto been identified [25,26,27]. Arsenic compounds have a rich history in medicine and have been shown to possess good therapeutic potentials against protozoan diseases such as African trypanosomiasis [5,28]. In addition, arsenic derivatives are currently explored for the treatment of cancer such as leukemia [29,30,31]. In general, compounds that are able to suppress cancer growth often also exhibit activities against intraerythrocytic proliferation, as has for example been shown for proteasome inhibitors or inhibitors preventing epigenetic regulation (e.g., [17,18,19,32,33]).

In this study, we show that organoarsenic compounds effectively act on intraerythrocytic growth and gametocyte development as well as gametogenesis of *P. falciparum*. These results confirm previous findings that organoasrsenic could be exploited in treating protozoa diseases [5,28]. Interestingly, the compounds did not exhibit any cytotoxic effect both on non-infected erythrocytes as well as on the leukemia Nalm-6 cell line indicating that the compounds are safe and mainly target the parasite. Additionally, the active compounds showed high activity against CQ-sensitive and CQ-resistant strains of *P. falciparum* suggesting that compounds target similar pathways in both CQ-resistant and CQ-sensitive strains.

The activity against the intraerythrocytic stages of *P. falciparum* was particularly evident for the early ring and trophozoite stages, suggesting that rather the metabolic pathways required for growth, but not the cell cycle machinery, would be targeted by the compounds. In addition, the active compounds were effective on developing gametocytes as well as on mature gametocytes that were prohibited to initiate gametogenesis. The fact that the compounds exhibit inhibitory effects both on the asexual blood stages as well as the sexual blood stages make them potential multi-stage drugs. The exact mode of action of the organoarsenic compounds as well as its potential action on other life-cycle stages of the malaria parasite has to be elucidated in future studies.

The most active compound, As-8, exhibited high activities against CQ-sensitive and CQ-resistant *P. falciparum*, in the low micromolar range. Noteworthy and contrary to the other compounds, As-8 possesses two naphthyl groups which could be important for its high activity. Hydroxy-substituted naphthalene compounds have also been shown to display strong antimalarial activity when tested against non-human malaria [34]. As-8 also showed the highest effect in the inhibition of gametocyte development and exflagellation, making it also a good transmission blocking agent. We therefore propose that As-8 will provide a lead structure for the further design of highly active organoarsenics with both asexual blood stage and transmission blocking activity.

## 5. Conclusions

The study reports the syntheses and evaluation of the antiplasmodial and transmission-blocking activity of novel organoarsenic compounds. Our data show that five out of the 11 synthesized compounds exhibit activities against the CQ-sensitive strains of *P. falciparum* with IC_50_ values ranging between 1.52 and 8.64 µM. In addition, the five active compounds exhibited an inhibitory effect against the CQ-resistant strain of *P. falciparum* as well as gametocyte development and exflagellation which are important for malaria transmission to the mosquito. Compound As-8 exhibited the highest potential to impair blood stage growth and parasite transmission, making it a good lead for the design of novel organoarsenic drugs with combined antimalarial and transmission blocking activity against the malaria parasite *P. falciparum*.

## Figures and Tables

**Table 1:** Antiplasmodial activity of organoarsenic compounds against the CQ- sensitive 3D7 and CQ-resistant Dd2 strains of *P. falciparum*.

Compounds	IC_50_ [µM]	IC_90_ [µM]	CC_50_ [µM]	SI
CQ-sensitive 3D7				
CQ	0.002 ± 0.0003	0.0189		
As-1	-	-		
**As-2**	**8.63 ± 0.606**	**77.67**	**>100**	**>12**
As-3	-	-		
**As-4**	**8.64 ± 0.963**	**77.76**	**>100**	**>12**
As-5	16.57 ± 3.092	149.13		
**As-6**	**7.55 ± 0.580**	**67.95**	**>100**	**>13**
**As-7**	**7.64 ± 0.387**	**68.76**	**>100**	**>13**
**As-8**	**1.52 ± 0.089**	**13.68**	**>100**	**>66**
As-9	45.65 ± 0.435	410.85		
As-10	18.13 ± 2.996	163.17		
As-11	43.04 ± 5.700	387.36		
CQ-resistant Dd2				
CQ	0.28 ± 0.022	2.52		
**As-2**	**7.88 ± 0.261**	**70.92**	**>100**	**>13**
As-4	30.05 ± 2.071	270.45	>100	>3
**As-6**	**8.27 ± 1.330**	**74.43**	**>100**	**>12**
As-7	34.92 ± 2.120	314.28	>100	>3
**As-8**	**0.35 ± 0.099**	**3.15**	**>100**	**>286**

CC_50_, half-maximal cytotoxic concentration; IC_50_, half-maximal inhibitory concentration; SI, selectivity index (ratio of CC_50_ and IC_50_); CQ, chloroquine. Bold indicate highly active compounds.

## Supplementary Materials

Supplementary materials can be found in Supplementary File 1 at https://www.mdpi.com/2227-9059/8/8/260/s1.

## Abbreviations

CQ	Chloroquine
IC_50_	Half maximal inhibitory concentration
IC_90_	90% inhibitory concentration
RT	Room temperature
DMSO	Dimethyl sulfoxide
APAD	3-Acetylpyridine adenine dinucleotide
LDH	Lactate dehydrogenase
NBT	Nitro Blue Tetrazolium
TLCK	Tosyl-l-lysyl-chloromethane hydrochloride
INT	2-[4-idophenyl]-3-[4-nitrophenyl]-5-phenyltetrazolium chloride
SI	Selectivity index

## References

[1] World Health Organization (2019). World Malaria Report 2019.

[2] Wadi I., Nath M., Anvikar A.R., Singh P., Sinha A. (2019). Recent advances in transmission-blocking drugs for malaria elimination. Future Med. Chem..

[3] Grund S.C., Hanusch K.S., Wolf H.W. (2008). Arsenic and arsenic compounds. Ullmann’s Encycl. Ind. Chem..

[4] Hughes M.F., Beck B.D., Chen Y., Lewis A.S., Thomas D.J. (2011). Arsenic Exposure and Toxicology: A Historical Perspective. Toxicol. Sci..

[5] Gibaud S., Jaouen G. (2010). Arsenic-based drugs: From fowler’s solution to modern anticancer chemotherapy. Top. Organomet. Chem..

[6] Flora S.J.S. (2015). Arsenic: Chemistry, Occurrence, and Exposure. Handbook of Arsenic Toxicology.

[7] Rebecca V.W., Nicastri M.C., Fennelly C., Chude C.I., Barber-Rotenberg J.S., Ronghe A., Mcafee Q., Mclaughlin N.P., Zhang G., Goldman A.R. (2019). PPT1 promotes tumor growth and is the molecular target of chloroquine derivatives in cancer. Cancer Discov..

[8] Nzila A., Okombo J., Becker R.P., Chilengi R., Lang T., Niehues T. (2010). Anticancer agents against malaria: Time to revisit?. Trends Parasitol..

[9] Doak G.O., Steinman H.G., Eagle H. (1941). The preparation of phenylarsenoxides. IV. Disubstituted compounds. J. Am. Chem. Soc..

[10] Jiang X.D., Matsukawa S., Kojima S., Yamamoto Y. (2012). Synthesis and characterization of antiapicophilic arsoranes and related compounds. Inorg. Chem..

[11] Jacobs W.A., Heidelberger M. (1919). The isomeric hydroxyphenylarsonic acids and the direct arsenation of phenol. J. Am. Chem. Soc..

[12] Holmes R.R., Day R.O., Sau A.C. (1995). Synthesis and molecular structure of spiroarsoranes differing in ring unsaturation. Distortion coordinates for five-coordinated arsenic1. Phosphorus. Sulfur. Silicon Relat. Elem..

[13] Kwong F.Y., Lai C.W., Yu M., Tan D.M., Lam F.L., Chan A.S., Chan K.S.C. (2005). Convenient Palladium-Catalyzed Arsination:  Direct Synthesis of Functionalized Aryl Arsines, Optically Active as, N Ligands, and Their Metal Complexes. Organometallics.

[14] Wahl G., Kleinhenz D., Schorm A., Sundermeyer J., Stowasser R., Rummey C., Bringmann G., Fickert C., Kiefer W. (1999). Peroxomolybdenum Complexes as Epoxidation Catalysts in Biphasic Hydrogen Peroxide Activation: Raman Spectroscopic Studies and Density Functional Calculations. Chem. A Eur. J..

[15] Ifediba T., Vanderberg J.P. (1981). Complete in vitro maturation of Plasmodium falciparum gametocytes. Nature.

[16] Lambros C., Vanderberg J.P. (1979). Synchronization of Plasmodium falciparum Erythrocytic Stages in Culture. J. Parasitol..

[17] Aminake M.N., Schoof S., Sologub L., Leubner M., Kirschner M., Arndt H.D., Pradel G. (2011). Thiostrepton and derivatives exhibit antimalarial and gametocytocidal activity by dually targeting parasite proteasome and apicoplast. Antimicrob. Agents Chemother..

[18] Ngwa C.J., Kiesow M.J., Papst O., Orchard L.M., Filarsky M., Rosinski A.N., Voss T.S., Llinás M., Pradel G. (2017). Transcriptional Profiling Defines Histone Acetylation as a Regulator of Gene Expression during Human-to-Mosquito Transmission of the Malaria Parasite Plasmodium falciparum. Front. Cell. Infect. Microbiol..

[19] Ngwa C.J., Kiesow M.J., Orchard L.M., Farrukh A., Llinás M., Pradel G. (2019). The g9a histone methyltransferase inhibitor BIX-01294 modulates gene expression during plasmodium falciparum gametocyte development and transmission. Int. J. Mol. Sci..

[20] Garcia G.E., Wirtz R.A., Barr J.R., Woolfitt A., Rosenberg R. (1998). Xanthurenic acid induces gametogenesis in Plasmodium, the malaria parasite. J. Biol. Chem..

[21] Billker O., Lindo V., Panico M., Etienne A.E., Paxton T., Dell A., Rogers M., Sinden R.E., Morris H.R. (1998). Identification of xanthurenic acid as the putative inducer of malaria development in the mosquito. Nature.

[22] Czesny B., Goshu S., Cook J.L., Williamson K.C. (2009). The proteasome inhibitor epoxomicin has potent Plasmodium falciparum gametocytocidal activity. Antimicrob. Agents Chemother..

[23] Rupp I., Bosse R., Schirmeister T., Pradel G. (2008). Effect of protease inhibitors on exflagellation in Plasmodium falciparum. Mol. Biochem. Parasitol..

[24] Sologub L., Kuehn A., Kern S., Przyborski J., Schillig R., Pradel G. (2011). Malaria proteases mediate inside-out egress of gametocytes from red blood cells following parasite transmission to the mosquito. Cell. Microbiol..

[25] Baragaña B., Hallyburton I., Lee M.C.S., Norcross N.R., Grimaldi R., Otto T.D., Proto W.R., Blagborough A.M., Meister S., Wirjanata G. (2015). A novel multiple-stage antimalarial agent that inhibits protein synthesis. Nature.

[26] Kato N., Comer E., Sakata-Kato T., Sharma A., Sharma M., Maetani M., Bastien J., Brancucci N.M., Bittker J.A., Corey V. (2016). Diversity-oriented synthesis yields novel multistage antimalarial inhibitors. Nature.

[27] Plouffe D.M., Wree M., Du A.Y., Meister S., Li F., Patra K., Lubar A., Okitsu S.L., Flannery E.L., Kato N. (2016). High-Throughput Assay and Discovery of Small Molecules that Interrupt Malaria Transmission. Cell Host Microbe.

[28] Fairlamb A.H. (2003). Chemotherapy of human African trypanosomiasis: Current and future prospects. Trends Parasitol..

[29] Chou W.C., Dang C.V. (2005). Acute promyelocytic leukemia: Recent advances in therapy and molecular basis of response to arsenic therapies. Curr. Opin. Hematol..

[30] Swindell E.P., Hankins P.L., Chen H., Miodragović C.D.S.U., O’Halloran T.V. (2013). Anticancer activity of small-molecule and nanoparticulate arsenic(III) complexes. Inorg. Chem..

[31] Cioloboc D., Kurtz D.M. (2020). Targeted cancer cell delivery of arsenate as a reductively activated prodrug. J. Biol. Inorg. Chem..

[32] Aminake M.N., Arndt H.-D.D., Pradel G. (2012). The proteasome of malaria parasites: A multi-stage drug target for chemotherapeutic intervention?. Int. J. Parasitol. Drugs Drug Resist..

[33] Trenholme K., Marek L., Duffy S., Pradel G., Fisher G., Hansen F.K., Skinner-Adams T.S., Butterworth A., Ngwa C.J., Moecking J. (2014). Lysine acetylation in sexual stage malaria parasites is a target for antimalarial small molecules. Antimicrob. Agents Chemother..

[34] Duffin W.M., Rollo I.M. (1957). Antimalarial activity of hydroxy-substituted naphthalene compounds. Br. J. Pharmacol. Chemother..

