# Supplementary material for: Organoarsenic Compounds with In Vitro Activity against the Malaria Parasite Plasmodium falciparum
Source: Biomedicines. 2020 Aug 2;8(8):260. doi: 10.3390/biomedicines8080260 (PMC7459655; doi:10.3390/biomedicines8080260)
Supplement: Supplementary file 1 [file biomedicines-08-00260-s001.pdf]

Organoarsenic compounds with *in-vitro* activity against the malaria parasite *Plasmodium falciparum*

Sofia Basova[1], Nathalie Wilke[2], Jan Christoph Koch[3], Aram Prokop[2,4], Albrecht Berkessel[3], Gabriele Pradel[1], Che Julius Ngwa[1*]

[1]Division of Cellular and Applied Infection Biology, Institute of Zoology, RWTH Aachen University, Worringerweg 1, 52074 Aachen, Germany

[2]Department of Paediatric Oncology, Children's Hospital Cologne, Amsterdamer Straße 59, 50735 Cologne, Germany

[3]Department of Chemistry, Organic Chemistry, University of Cologne, Greinstraße 4, 50939 Cologne, Germany

[4]Department of Paediatric Oncology, Helios Hospital Schwerin, Wismarsche Strasse 393-397, 19049 Schwerin, Germany

*Correspondence: ngwa.che@bio2.rwth-aachen.de

Supplementary Information 1: Synthesis and Characterization of Compound As-1

A 10 ml round-bottomed flask, equipped with a magnetic stir bar, was charged with a solution of 108 mg (500 µmol, 1.00 eq) *ortho*-arsanilic acid in 1.00 ml (1.00 mmol, 2.00 eq) 1 N aq. sodium hydroxide and cooled to 0 °C. At this temperature, 75.0 µl (590 µmol, 1.18 eq) (*S*)-2-acetoxypropionyl chloride were added. Over the course of the reaction, the pH of the mixture was kept alkaline by addition of 300 µl (600 µmol, 1.20 eq) 2 N aq. sodium hydroxide. After 1 h, the pH of the mixture was lowered to 1, by addition of conc. hydrochloric acid. A colorless precipitate occurred which was filtered off. The arsonic acid **As-1** (100 mg, 300 µmol, 60 %) was obtained as a colorless solid. Recrystallization from methanol afforded colorless needles suitable for X-ray crystallography.

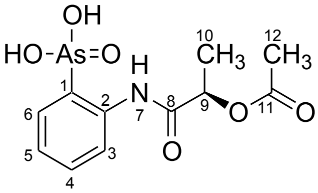

**As-1**	$C_{11}H_{14}AsNO_6$ (331.15 g/mol)
**Melting point**	Decomposition at 180 °C
**^{1}H-NMR**	(300 MHz, DMSO-d_6) δ = 11.73 (s; 1H, N7-H), 8.49 (d; ^3J = 7.98 Hz, 1H, C3-H), 7.65 (d; ^3J = 7.42 Hz, 1H, C6-H), 7.63 (dd; ^3J = 7.98 Hz, ^3J = 8.11 Hz, 1H, C4-H), 7.29 (dd; ^3J = 7.42 Hz, ^3J = 8.11 Hz, 1H, C5-H), 5.07 (d; ^3J = 7.01 Hz, 1H, C9-H), 2.18 (s; 3H, C12-H), 1.43 (d; ^3J = 7.01 Hz, 3H, C10-H)
**^{13}C-NMR**	(75.5 MHz, DMSO-d_6) δ = 183.8 (s; C11), 169.4 (s; C8), 149.2 (s; C2), 133.3 (d; C4), 132.8 (d; C6), 132.6 (s; C1), 119.5 (d; C5), 118.3 (d; C3), 69.7 (d; C9), 24.5 (q; C12), 21.4 (q; C10)
**FT-IR**	(KBr) $\tilde{\nu}$ [cm^{-1}] = 3221 (m), 1744 (s), 1692 (s), 1582 (w), 1541 (s), 1289 (w), 1233 (s), 1131 (w), 1103 (w), 1048 (w), 774 (s), 637 (w), 466 (w), 439 (w)
**Specific rotation**	$[\alpha]_D^{20}$ = -50 ° (c = 0.795, methanol)

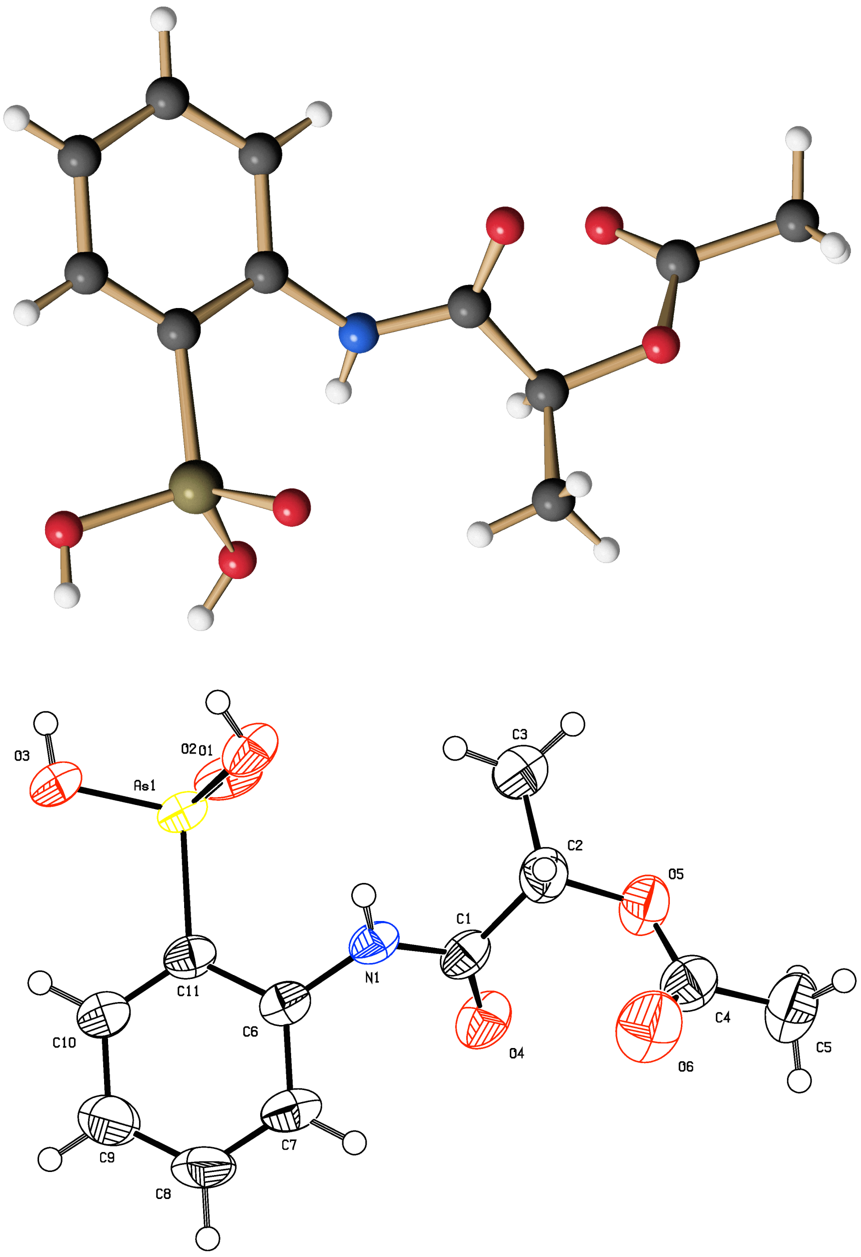

Figure S1: Molecular structure (top) and ORTEP (bottom) of the X-ray crystal structure of **As-1**. Thermal ellipsoids are drawn at 50% probability level; CCDC 2013348.

Supplementary Information 2: Synthesis and Characterization of Compound As-2

A 500 mL round-bottomed flask, equipped with a stir bar, was charged with 250 mL water. 2,6-Dimethylaniline (22.0 ml, 177 mmol, 1.00 eq) and 44.3 ml (443 mmol, 2.50 eq) conc. hydrochloric acid were added, and the resulting suspension was cooled to 0°C. With stirring, a solution of 13.5 g (195 mmol, 1.10 eq) sodium nitrite in 25.0 ml water was added to the suspension, while maintaining the temperature at 0 °C. After 20 min at 0 °C, the mixture was filtered at 0 °C, and the filtrate was added slowly, in a dropwise manner, to a stirred solution of 46.0 g (354 mmol, 2.00 eq) sodium meta-arsenite, 29.0 g (265 mmol, 1.50 eq) sodium carbonate, and 830 mg (3.54 mmol, 0.02 eq) copper(II) sulfate pentahydrate in 250 ml water, pre-heated to 80 °C. The reaction mixture was stirred at 80 °C for 1 h, cooled to room temperature, and filtered. The pH of the brown solution was adjusted to 1 by addition of conc. hydrochloric acid, resulting in foaming and the precipitation of a colorless solid. The solid was filtered off, affording 13.7 g (60.2 mmol, 34 %) of 2,6-dimethylphenylarsonic acid (**As-2**) as a brownish powder. Recrystallization from methanol afforded colorless needles suitable for X-ray crystallography.

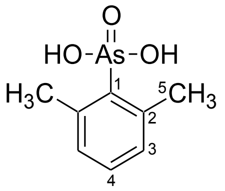

**As-2**	$C_8H_{11}AsO_3$ (230.09 g/mol)			
**Melting point**	>270 °C			
**CHN Analysis**		C [%]	H [%]	N [%]
	calculated	41.76	4.82	0.00
	found	41.52	4.87	0.00

^{1}H-NMR (300 MHz, DMSO-d_6) δ = 7.37 (d; ^3J = 7.57 Hz, 2H, C3-H), 7.16 (t; ^3J = 7.57 Hz, 1H, C4-H), 2.64 (s; 6H, C5-H)

^{13}C-NMR (75.5 MHz, DMSO-d_6) δ = 141.5 (s; C2), 132.3 (d; C4), 131.8 (s; C1), 129.6 (d; C3), 22.3 (q; C5)

FT-IR (KBr) $\tilde{\nu}$ [cm^{-1}] = 2816 (m), 2338 (m), 1577 (w), 1458 (s), 1390 (m), 1243 (w), 1190 (w), 1038 (w), 886 (s), 778 (s), 488 (w), 406 (m)

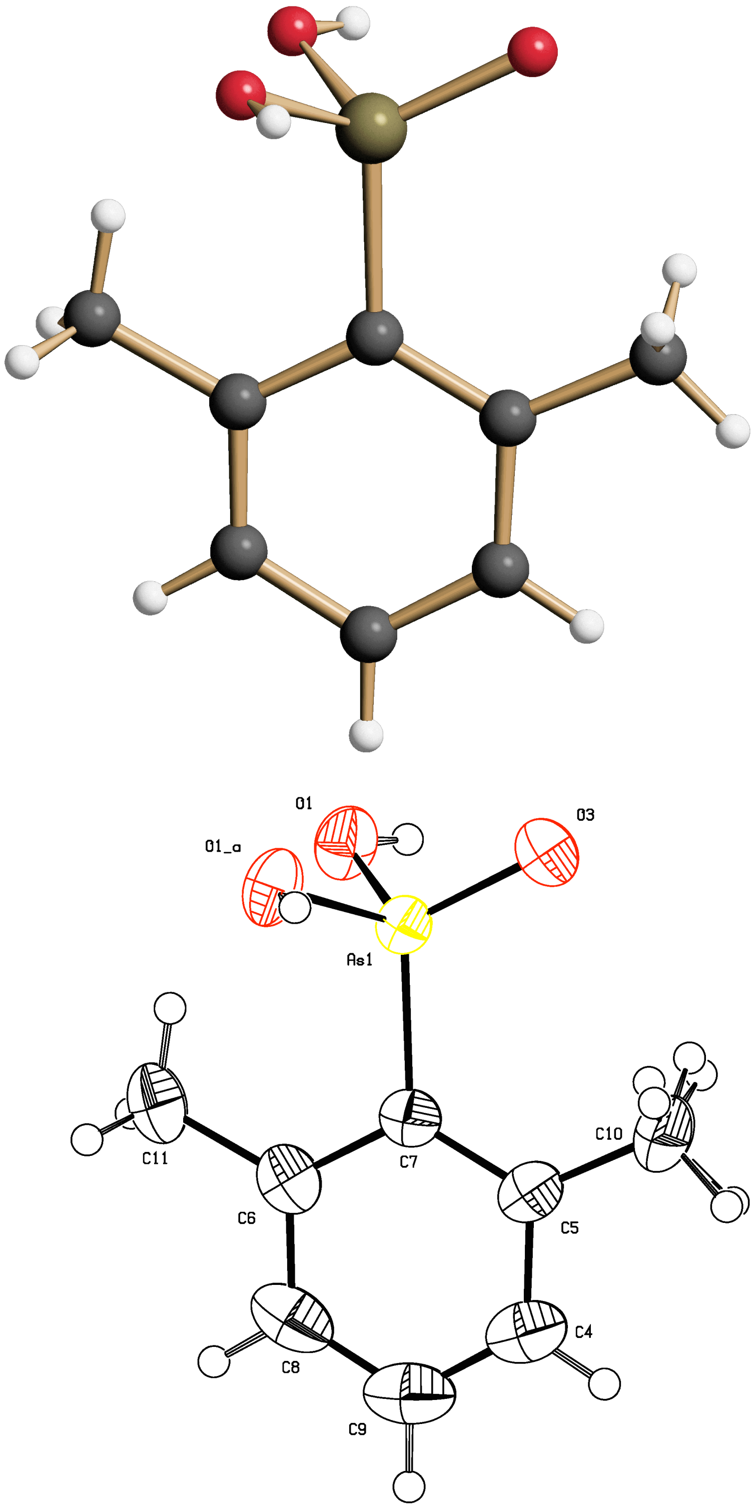

Figure S2: Molecular structure (top) and ORTEP (bottom) of the X-ray crystal structure of **As-2**. Thermal ellipsoids are drawn at 50% probability level; CCDC 2013345.

Supplementary Information 3: Synthesis and Characterization of Compound As-3

A 500 ml round-bottomed flask, equipped with a magnetic stir bar and a reflux condenser, was charged with a solution of 10.0 g (43.5 mmol, 1.00 eq) 2,6-dimethylphenylarsonic acid (**As-2**), 27.0 g (170 mmol, 4.00 eq) potassium permanganate und 5.73 g (100 mmol, 2.35 eq) potassium hydroxide in 200 ml water. The mixture was heated to reflux, with stirring, for 12 h. After cooling to room temperature, the brown manganese dioxide that had precipitated was filtered off. The filtrate was adjusted to neutral pH by addition of conc. hydrochloric acid, and was filtered again. The pH of the filtrate was then adjusted to 1 by addition of more hydrochloric acid. A colorless precipitate formed which was filtered off, affording 6.84 g (22.2 mmol, 51 %) of compound **As-3**, as a dihydrate, in the form of a colorless powder.

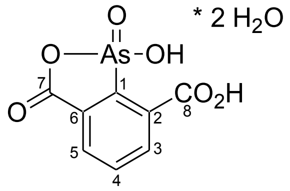

As-3 (dihydrate) $C_8H_9AsO_8$ (308.07 g/mol)

Melting point >250 °C

CHN Analysis

	C [%]	H [%]	N [%]
calculated	31.19	2.94	0.00
found	31.00	3.36	0.00

^{1}H-NMR (300 MHz, DMSO-d$_6$) δ = 8.08 (d; ^{3}J = 6.75 Hz, 1H, C5-H), 8.08 (d; ^{3}J = 8.08 Hz, 1H, C3-H), 7.95 (dd; ^{3}J = 6.75 Hz, ^{3}J = 8.08 Hz, 1H, C4-H)

^{13}C-NMR (75.5 MHz, DMSO-d$_6$) δ = 164.4 (s; C7 and C8), 140.8 (s; C1), 136.5 (d; C4), 133.2 (s; C2 and C6), 129.2 (d; C3 and C5)

FT-IR (KBr) $\tilde{\nu}$ [cm^{-1}] = 3575 (m), 3495 (m), 3083 (w), 1669 (s), 1288 (m), 1165 (m), 1149 (m), 1089 (s), 918 (m), 809 (m), 765 (m), 744 (m), 672 (m), 469 (m), 411 (w)

Supplementary Information 4: Synthesis and Characterization of Compound As-8

A 100 ml three-necked flask, equipped with a magnetic stir bar, a septum and a glass stopper was charged with a solution of 1.50 g (6.78 mmol, 1.00 eq.) 1-bromo-2-methylnaphthalene in 50.0 ml abs. diethyl ether under argon. The solution was cooled to -78 °C and *tert.*-butyllithium in *n*-pentane (1.5 M, 9.05 ml, 13.6 mmol, 2.00 eq.) was added through the septum by means of a syringe. The reaction mixture was stirred at -78 °C for 30 min. Arsenic trichloride (240 µl, 3.39 mmol, 0.50 eq.) was added through the septum by means of a syringe. The reaction mixture was allowed to warm to room temperature overnight, with stirring. To the resulting dark brown mixture, a pre-mixed solution of 2.00 g sodium bicarbonate in 20 ml water and 4.00 ml 35 % aqueous hydrogen peroxide was added. When the evolution of gas had ceased, the pH of the mixture was adjusted to 1 by addition of dilute hydrochloric acid. A colorless precipitate formed which was soluble neither in the organic, nor in the aqueous phase. This solid material was filtered off, and washed with both water and diethyl ether. Recrystallization from ethanol afforded 1.00 g (2.57 mmol, 38 %) of bis-(2-methylnaphthalen-1-yl)-arsinic acid **As-8** as colorless crystals.

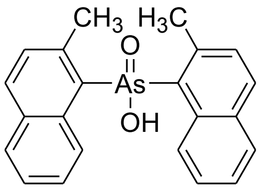

**As-8**	$C_{22}H_{19}AsO_2$; 390.31 g/mol
**Melting point**	292 °C, decomposition
**^{1}H-NMR**	(300 MHz, CD_3OD) δ = 2.57 (s; 6H, Me), 7.35-7.55 (m; 6H, aromatic H), 7.88-7.95 (m; 2H, aromatic H), 7.98-8.05 (m; 2H, aromatic H), 9.10-9.18 (m; 2H, aromatic H)
**^{13}C-NMR**	(75 MHz, CD_3OD) δ = 22.6 (q; 2C, Me), 126.1 (s; 2C, aromatic C), 127.4 (d; 2C, aromatic CH), 128.9 (d; 2C, aromatic CH), 130.1 (d; 2C, aromatic CH), 130.8 (d; 2C, aromatic CH), 131.2 (d; 2C, aromatic CH), 134.2 (d; 2C, aromatic CH), 134.6 (s; 2C, aromatic C), 134.9 (s; 2C, aromatic C), 143.5 (s; 2C, aromatic C)
**ESI-MS**	m/z (%, formula): 389.05 (100, $[(M\text{-}H)^-]$: $^{12}C_{22}H_{18}AsO_2$)

**CHN Analysis**	calculated:	C: 67.70 %	H: 4.91 %	N: 0.00 %
	found:	C: 67.66 %	H: 4.89 %	N: 0.00 %

**FT-IR**	(KBr) $\tilde{\nu}$ [cm^{-1}] = 3442 [br], 3060, 3007, 2924 [all w], 2690 [br], 2361 [m], 1620, 1505, 1445, 1423, 1357, 1321 [all m], 1203, 1166, 1149 [all w], 1031, 972 [both m], 876, 818, 770, 749 [s], 643, 524, 524, 486 [all w], 416 [s]

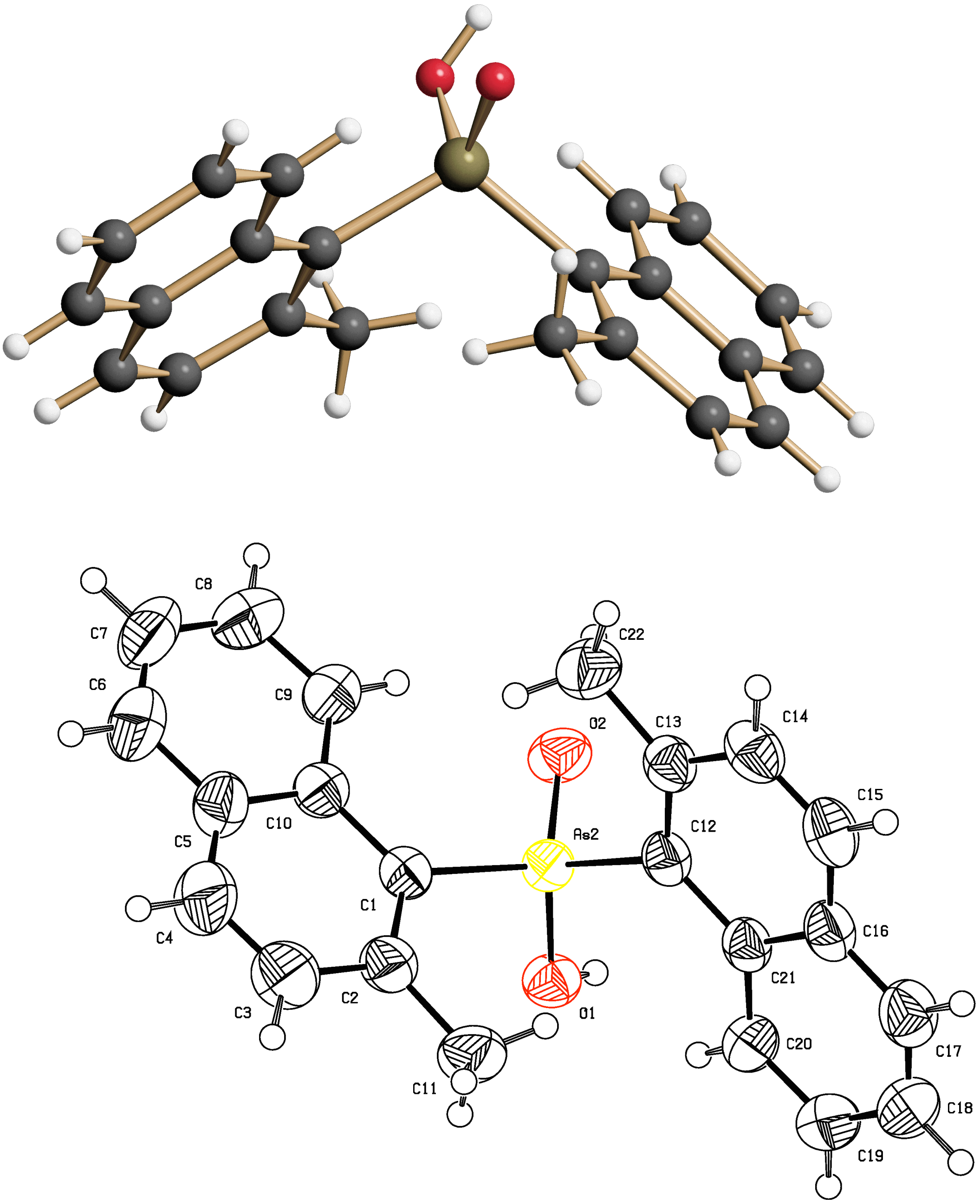

Figure S3: Molecular structure (top) and ORTEP (bottom) of the X-ray crystal structure of **As-8**. Thermal ellipsoids are drawn at 50% probability level; CCDC 2013346.

Supplementary Information 5: Synthesis and Characterization of Compound As-9

In a 100 ml three-necked flask, equipped with a magnetic stir bar, a rubber septum and an argon bubbler, 122 mg (3.04 mmol, 1.47 eq.) potassium hydride in mineral oil were washed with abs. diethyl ether. A solution of 2-bromo-3,5-di-*tert.*-butylbenzoic acid (865 mg, 2.76 mmol, 1.34 eq.) in 50 ml abs. diethyl ether was added to the hydride, and the mixture was stirred for ca. 15 min at room temperature. When the evolution of gas had ceased, the mixture was cooled to –78 °C, and *tert.*-butyllithium in *n*-pentane (1.5 M, 3.68 ml, 5.52 mmol, 2.67 eq.) was added through the septum by means of a syringe. The brown reaction mixture was stirred at -78 °C for 15 min. Arsenic trichloride (175 µl, 375 mg, 2.07 mmol, 1.00 eq.) was added through the septum by means of a syringe. The reaction mixture was allowed to warm to room temperature overnight, with stirring. To the resulting dark brown mixture, a pre-mixed solution of 1.50 g sodium bicarbonate in 30 ml water and 6.00 ml 35 % aqueous hydrogen peroxide was added. When the evolution of gas had ceased, the pH of the mixture was adjusted to 1 by addition of dilute hydrochloric acid. The organic phase was separated, dried over anhydrous magnesium sulfate, and rota-evaporated. Recrystallization of the crude product from methanol gave 235 mg (691 µmol, 33 %) of the anhydride **As-9** as colorless needles.

As-9 $C_{15}H_{21}AsO_4$; 340.25 g/mol

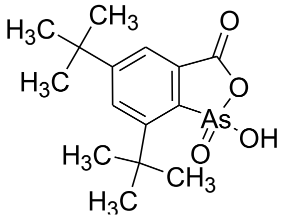

Melting point 255 °C, decomposition

^{1}H-NMR (300 MHz, $CDCl_3$) δ = 1.38 (s; 9H, ^{t}Bu), 1.61 (s; 9H, ^{t}Bu), 8.00 (d, $^4J_{H-H}$ = 1.8 Hz; 1H, aromatic H), 8.14 (d, $^4J_{H-H}$ = 1.8 Hz; 1H, aromatic H), 8.71 (s; 1H, OH)

^{13}C-NMR (75 MHz, $CDCl_3$) δ = 31.0 (q; 3C, ^{t}Bu), 31.6 (q; 3C, ^{t}Bu), 35.8 (s; 1C, ^{t}Bu), 36.6 (s; 1C, ^{t}Bu), 124.1 (s; 1C, aromatic C), 124.2 (d; 1C, aromatic CH), 132.0 (d; 1C, aromatic CH), 132.4 (s; 1C, aromatic C), 154.5 (s; 1C, aromatic C), 159.9 (s; 1C, aromatic C), 162.0 (s; 1C, C=O)

**CHN Analysis**	calculated:	C: 52.96 %	H: 6.22 %	N: 0.00 %
	found:	C: 52.56 %	H: 6.30 %	N: 0.00 %

FT-IR (KBr) $\tilde{\nu}$ [cm^{-1}] = 2970 [m], 1764 [s], 1600, 1480, 1367 [all w], 1236 [s], 1180, 1101 [both m], 942 , 896 [both s], 833 [m], 785 [s]

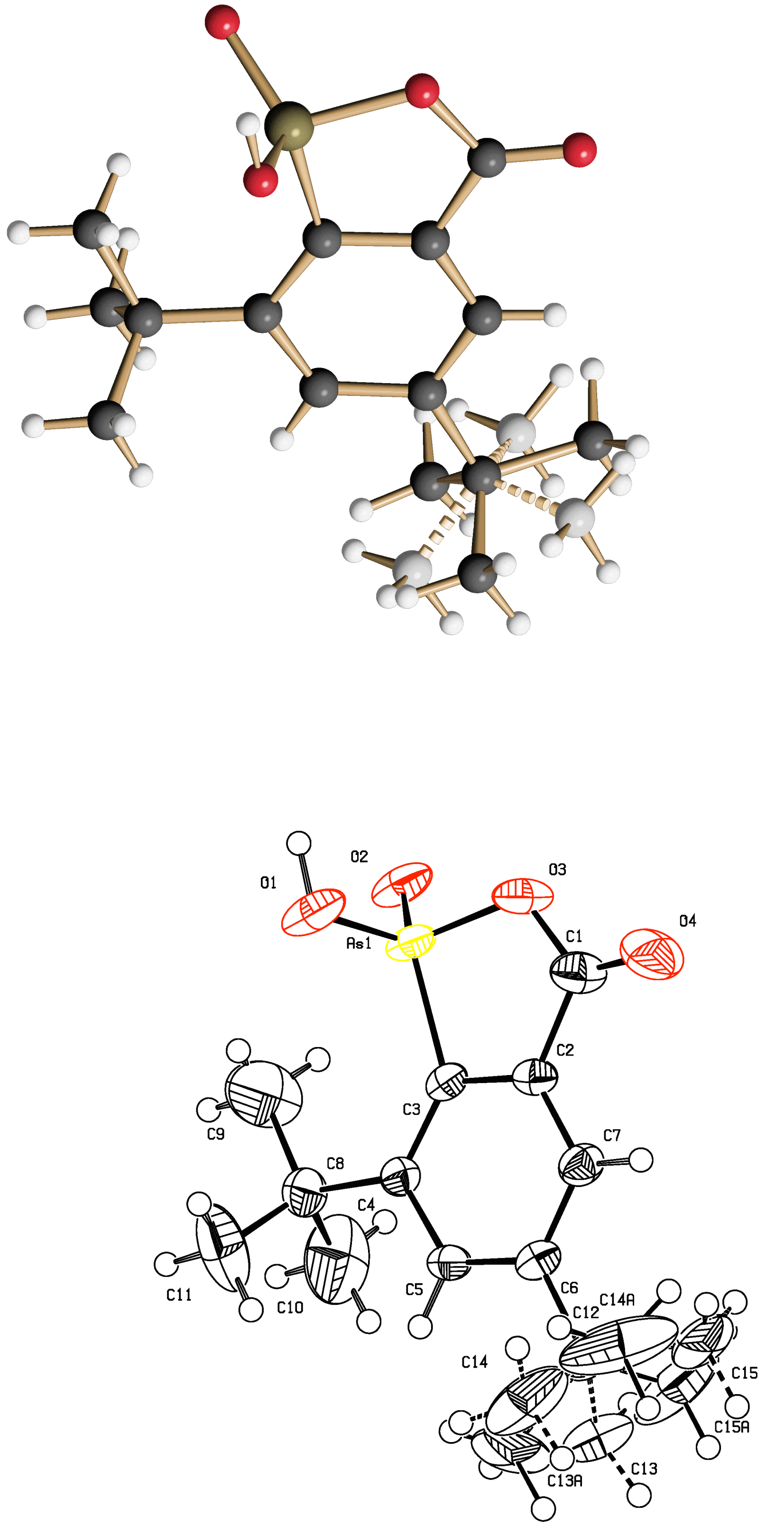

Figure S4: Molecular structure (top) and ORTEP (bottom) of the X-ray crystal structure of **As-9**. Thermal ellipsoids are drawn at 50% probability level; CCDC 2013347.

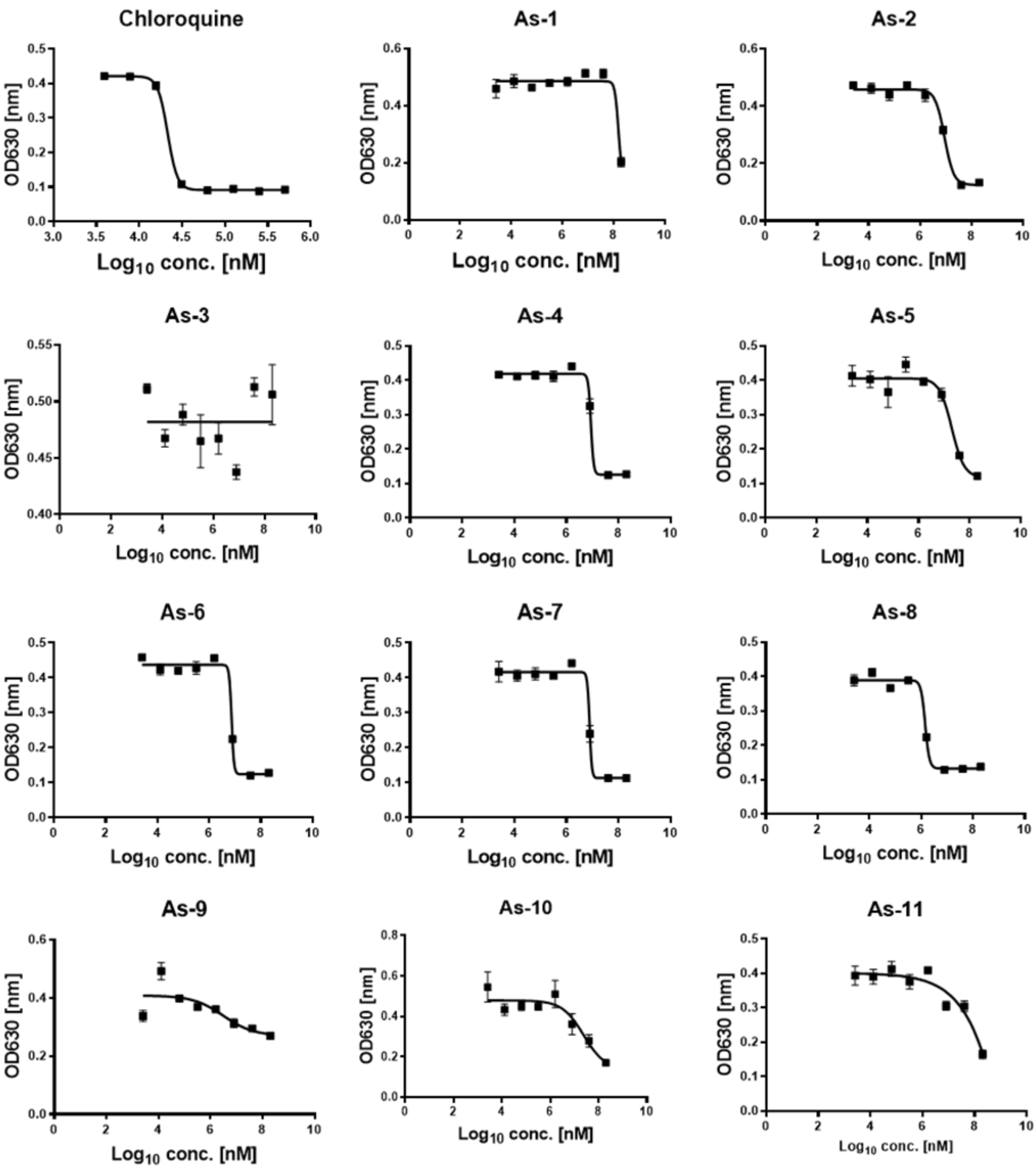

Figure S5. Logarithmic dose response curves for the testing of the organoarsenic compounds on the *P. falciparum* 3D7 strain. Ring stage cultures were incubated with the compounds for 72 h at 37°C at concentrations of 2.6 nM to 200 µM. CQ served as control. The viability of the parasites was assessed by plasmodial LDH activity using the Malstat assay, and the absorbance of the photometric reaction was measured at $OD_{630\,nm}$. The data are representative of three to five independent experiments.

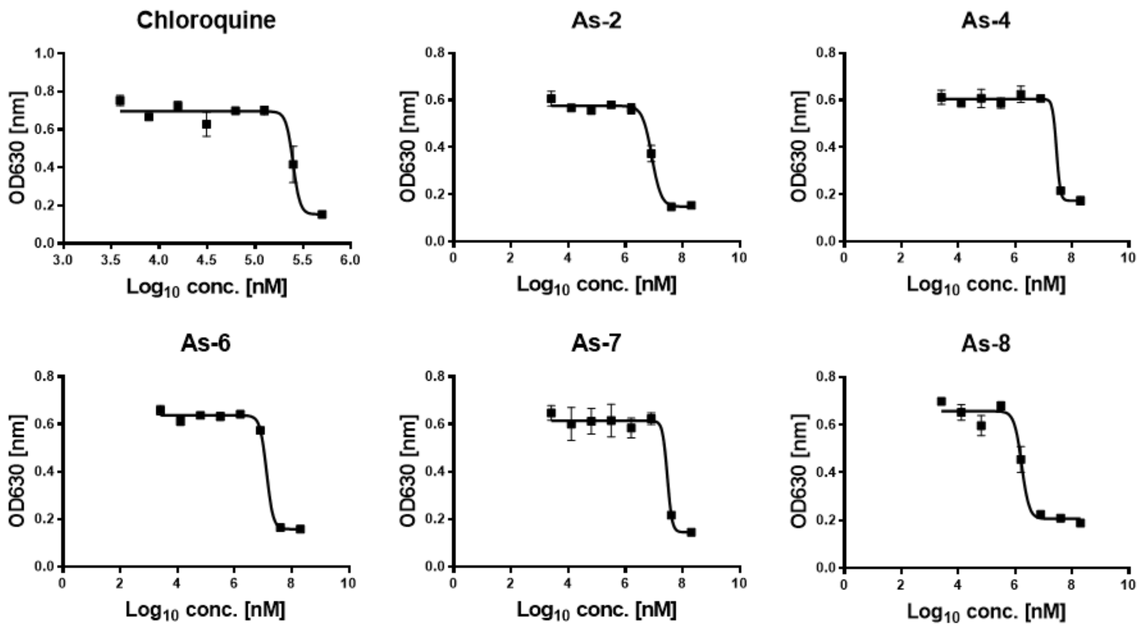

Figure S6. Logarithmic dose response curves for the testing of the organoarsenic compounds on the Dd2 strain. Ring stage cultures were incubated with the compounds for 72 h at 37°C at concentrations ranging from 200 μM to 2.6 nM. CQ served as control. The viability of the parasites was assessed using the Malstat assay, and the absorbance of the reaction was measured at $OD_{630\ nm}$. The data are representative of three to five independent experiments.

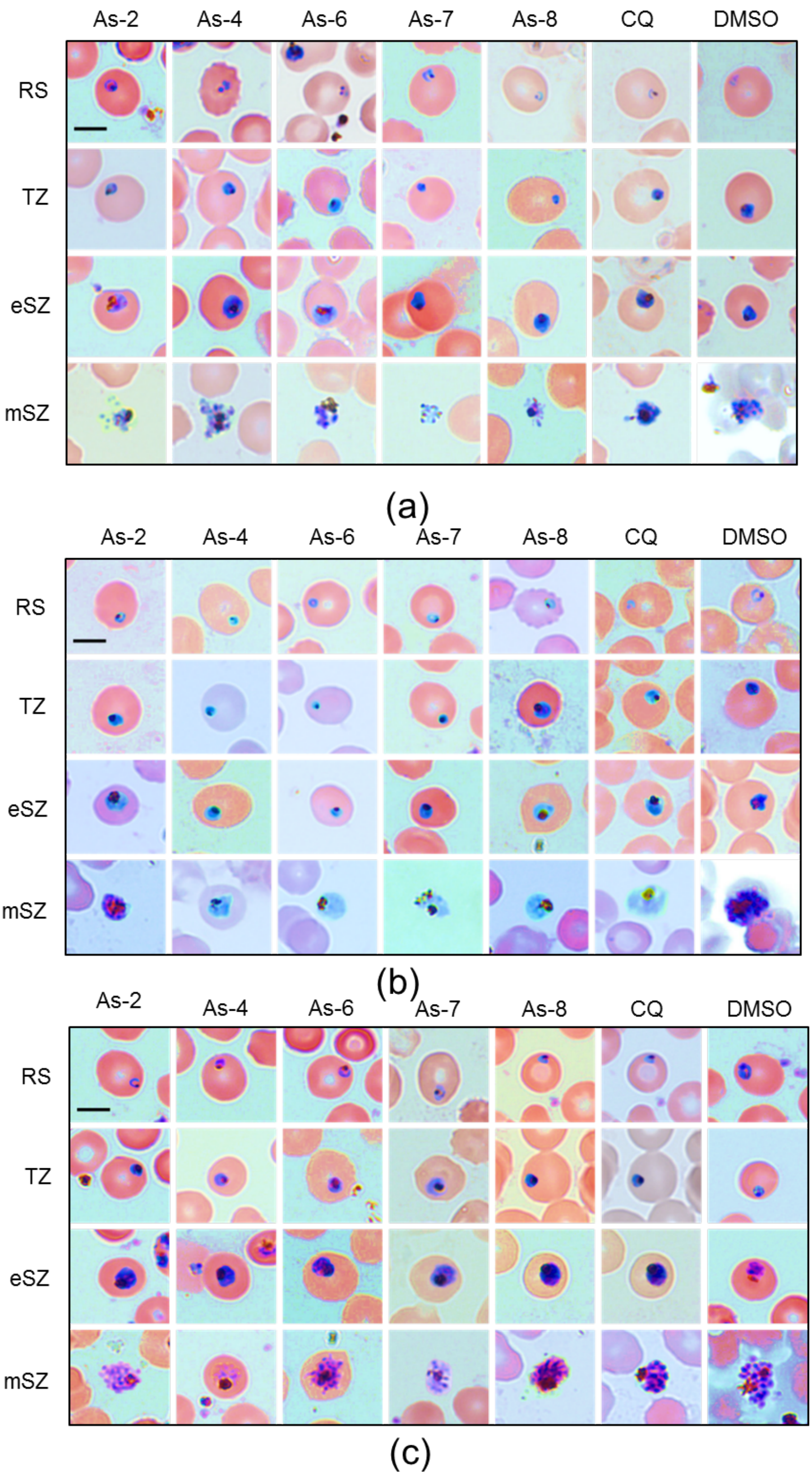

Figure S7. Parasite morphology after addition of organoarsenic compounds. Ring stages (a), trophozoites (b) and schizonts (c) were incubated with the compounds at IC_{90} concentrations for 48 h at 37°C and Giemsa smears were prepared. RS, ring stage; TZ, trophozoite; eSZ, early schizont; m SZ, mature schizont. Bar, 5 μm.

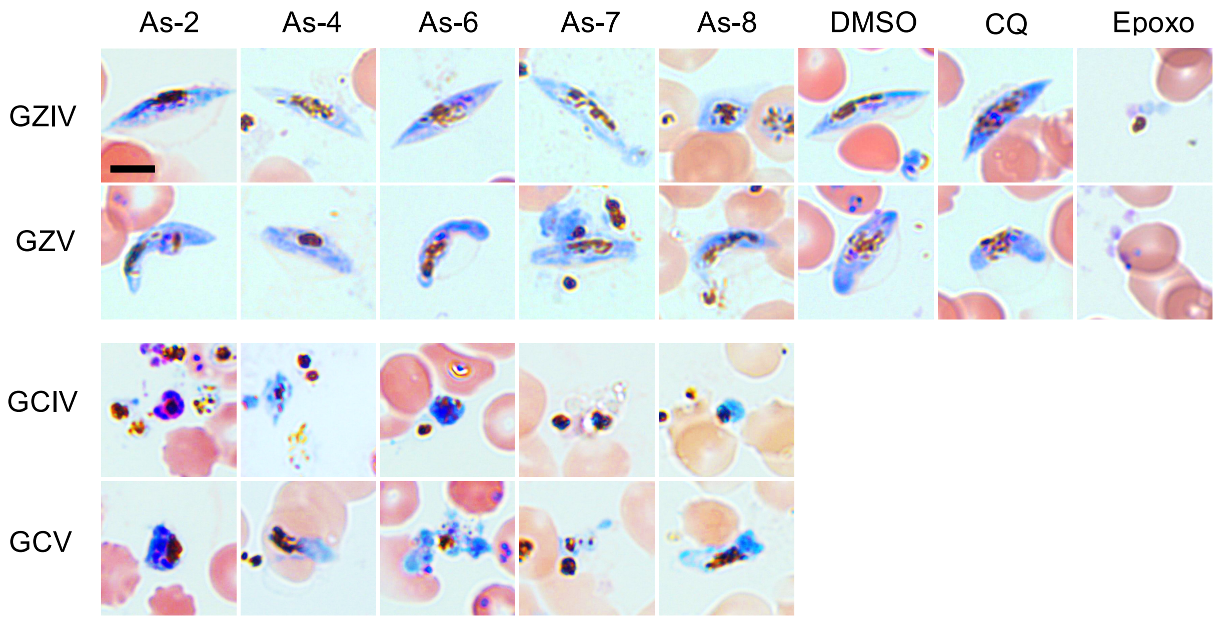

Figure S8. Gametocyte morphology after addition of organoarsenic compounds. Stage II gametocytes were incubated with the compounds at IC_{50} and IC_{90} concentrations for 2 d and further cultivated for 5 d at 37°C and Giemsa smears were prepared. GCIV, gametocyte stage IV; GCV, gametocyte stage V. Bar, 5 µm.

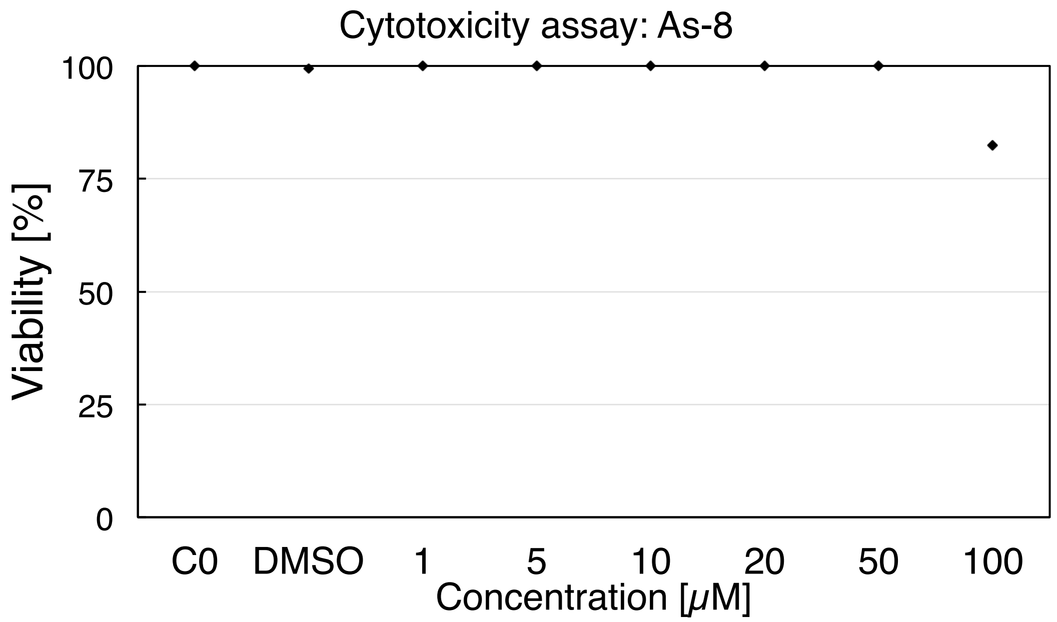

Figure S9. Cytotoxic effect of organoarsenic compounds. Human Nalm-6 cell were incubated with As-8 for 1 h at 37°C at concentrations of 1 to 100 µM. DMSO and cell culture medium (C0) served as control. The viability of the cells was assessed by potential LDH release, and the absorbance of the photometric reaction was measured at $OD_{492\ nm}$. The data are representative of three independent experiments.
